# Atypical Presentation of Inferior Vena Cava Thrombosis Induced by Recreational Cliff Diving Trauma

**DOI:** 10.7759/cureus.86581

**Published:** 2025-06-23

**Authors:** Salman B Syed, Harishankar Gopakumar, Tulika Chatterjee, Abuzar A Asif, Sonu Dhillon

**Affiliations:** 1 Internal Medicine, University of Illinois College of Medicine, Peoria, USA; 2 Internal Medicine, OSF Saint Francis Medical Center, Peoria, USA; 3 Gastroenterology, University of Illinois College of Medicine, Peoria, USA

**Keywords:** cliff diving, deep vein thrombosis (dvt), dvt, inferior vena cava thrombosis, ivct, ivc thrombosis, retroperitoneal hematoma, retroperitoneal mass

## Abstract

Inferior vena cava thrombosis (IVCT) is often underdiagnosed as it is not typically considered a primary diagnosis. Etiologies of IVCT can be broadly categorized into congenital and acquired causes, with the latter being more prevalent. Among acquired causes, blunt trauma, though uncommon due to the retroperitoneal location and elasticity of the inferior vena cava (IVC), can lead to thrombosis, particularly when involving the retrohepatic or intrapericardial segments. The liver's size and intraperitoneal position make it susceptible to deceleration injuries that may shear the IVC. A retroperitoneal hematoma can lead to IVCT through a combination of venous stasis, direct endothelial injury, and trauma-induced coagulopathy, the classic components of Virchow's triad.

We report a rare case of IVCT secondary to retroperitoneal hematoma caused by blunt trauma sustained during cliff diving in a previously healthy 19-year-old male. A comprehensive review of PubMed, Google Scholar, and Medline suggests this is the first reported instance of IVCT attributed to sport-induced shearing forces.

## Introduction

The inferior vena cava (IVC) is the largest vein in the human body, responsible for returning deoxygenated blood from the lower extremities, pelvis, and abdominal organs to the right atrium of the heart [1]. It plays a critical role in maintaining venous return and systemic hemodynamics, thereby contributing to adequate cardiac output and blood pressure regulation. Anatomically, the IVC is located in the retroperitoneal space, posterior to the abdominal viscera [1,2].

The IVC is subdivided into four anatomical segments: infrarenal, suprarenal, renal, and hepatic [1-3]. Each segment may be affected differently by pathological processes, including trauma, neoplastic invasion, or thrombus formation.

Inferior vena cava thrombosis (IVCT) refers to the formation of a thrombus within the lumen of the IVC. Though relatively uncommon, IVCT carries significant clinical implications and is considered part of the spectrum of deep venous thrombosis (DVT) [1]. It remains underdiagnosed and is frequently associated with substantial morbidity and mortality, with a reported mortality rate nearly twice that of lower extremity DVT [4].

The clinical presentation of IVCT is often variable and nonspecific, which can complicate timely diagnosis. If left untreated, IVCT may result in serious sequelae. These include post-thrombotic syndrome in up to 90% of cases, characterized by chronic venous insufficiency, leg swelling, pain, and skin changes; venous claudication in approximately 45%, manifesting as exertional leg discomfort due to impaired venous return; pulmonary embolism in about 30%, caused by embolic migration of thrombus to the pulmonary arteries; and venous ulceration in 15%, resulting from chronically elevated venous pressures [4].

IVCT is broadly categorized into congenital and acquired types [4,5]. While most congenital IVC anomalies (e.g., IVC agenesis or duplication) remain clinically silent due to collateral venous development, acquired IVCT may result from spontaneous thrombosis, external compression (e.g., by tumors or fibrosis), or direct endothelial injury [5].

Treatment is etiology-dependent, with anticoagulation as first-line therapy. Selected patients may require thrombolysis, thrombectomy, or stenting [4,5].

## Case presentation

A 19-year-old male presented to the emergency department with a one-month history of worsening dull low back pain that radiated to the tailbone and groin, exacerbated by physical activity. There was no history of trauma, fever, incontinence, unexplained weight loss, long-term steroid use, or parenteral drug abuse. No personal or family history of malignancy or thrombophilia was noted.

He was afebrile with a normal heart rate and blood pressure. Physical examination was notable for bilateral lower extremity swelling in the calf with no tenderness, erythema, or local warmth. Complete blood count, metabolic panel, and prothrombin time were within the normal range.

**Figure 1:**
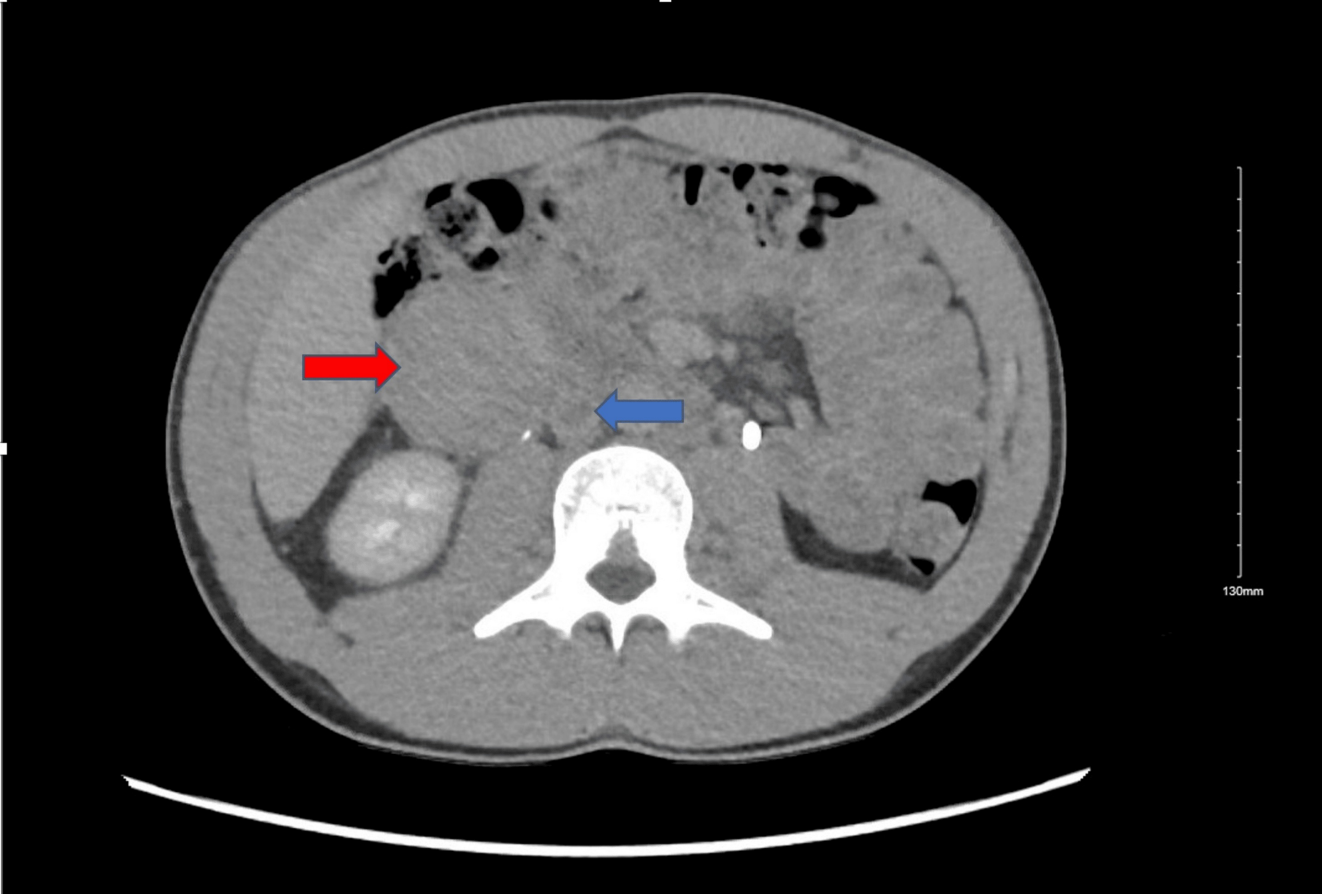
CT of the abdomen demonstrating a large right retroperitoneal mass measuring 7.5 cm (red arrow) and venous thrombus throughout the infrarenal inferior vena cava (blue arrow).

**Figure 2:**
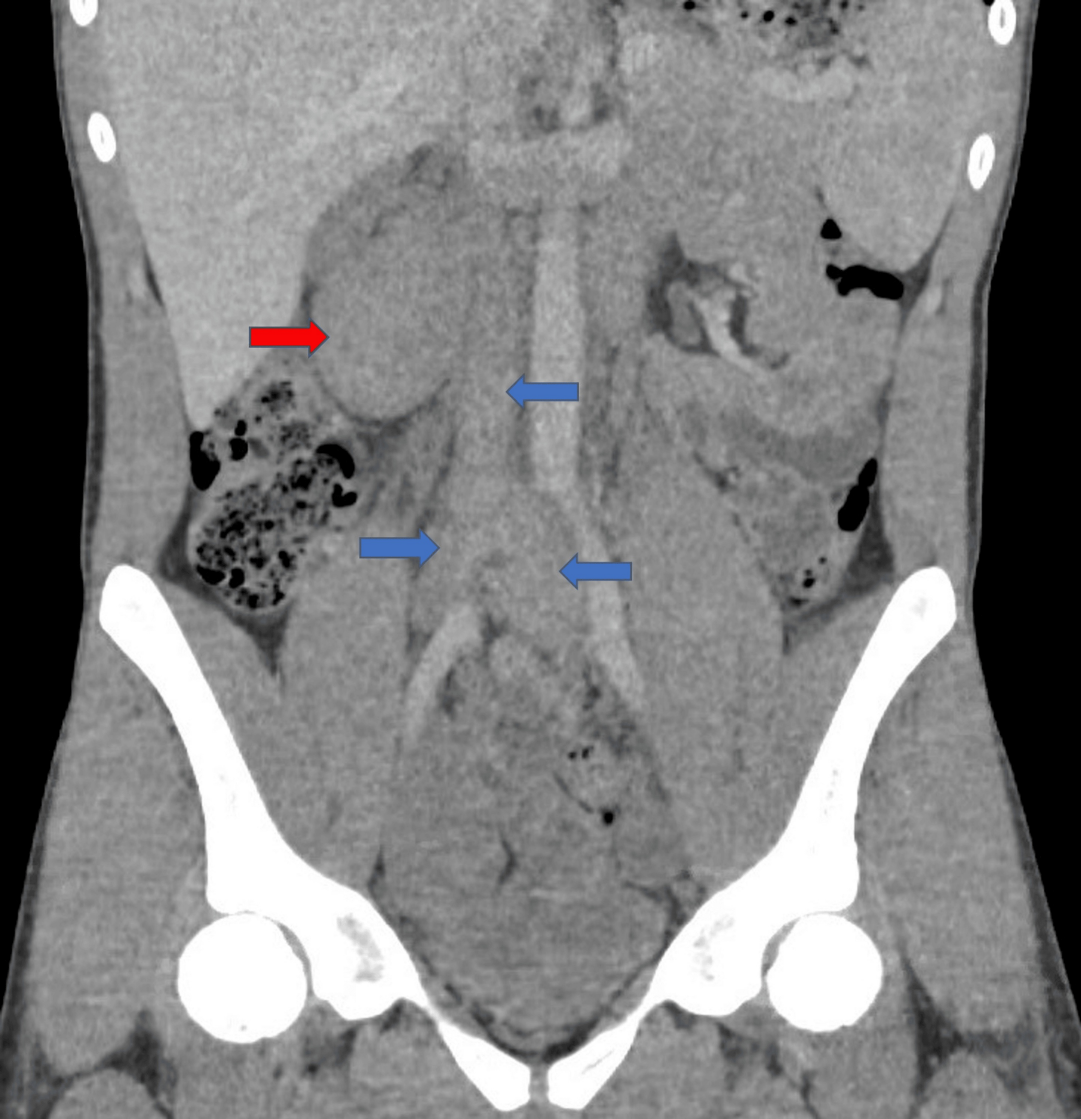
CT of the abdomen and pelvis demonstrating a large retroperitoneal mass (red arrow) and venous thrombus throughout the infrarenal inferior vena cava, and common right and left iliac veins (blue arrows).

CT of the abdomen and pelvis showed multiple right retroperitoneal soft tissue masses (largest 7.5 cm) and thrombus in the infrarenal IVC, common iliac, and common femoral veins (Figures 1, 2). Lower extremity venous duplex showed thrombosed bilateral iliac veins but no lower extremity DVT. CT angiography of the chest ruled out pulmonary embolism. Given these findings, anticoagulation with heparin drip was initiated.

**Figure 3:**
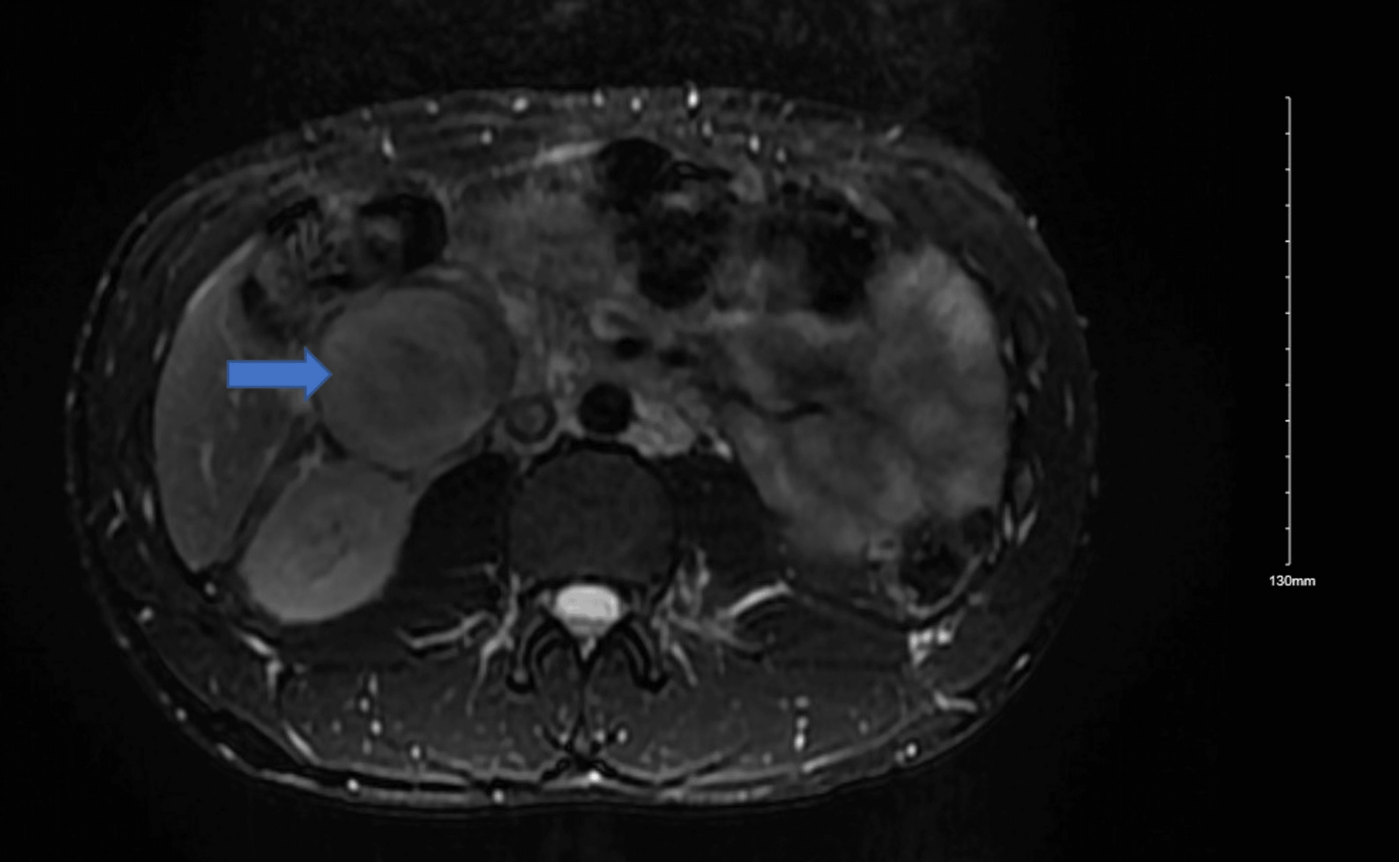
MRI of the abdomen demonstrating a retroperitoneal mass compressing the infrarenal inferior vena cava (blue arrow).

MRI of the abdomen and pelvis with contrast confirmed venous thrombosis and compression of the IVC by a mass (Figure 3). Endoscopic ultrasound (EUS) identified a 5.7 × 4.7 cm cystic-solid retroperitoneal mass adjacent to the duodenum/pancreas, compressing the IVC. EUS-guided fine needle aspiration (FNA) revealed only blood with no malignant cells.

**Figure 4:**
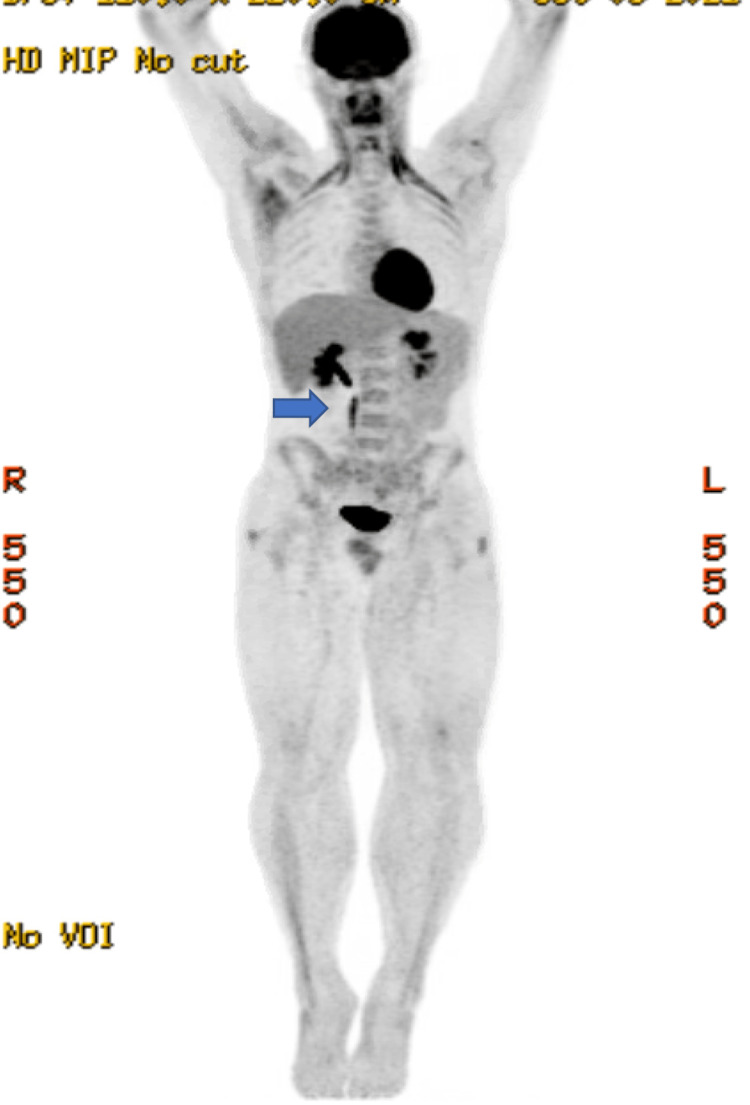
Positron emission tomography shows the dominant mass (blue arrow) in the right hemiabdomen is devoid of fluorodeoxyglucose (FDG) uptake.

Positron emission tomography (PET) scan obtained prior to EUS-guided FNA showed no fluorodeoxyglucose (FDG) uptake within the lesion, further lowering suspicion of malignancy (Figure 4). The complete lack of FDG uptake within this mass-like structure and the lack of evidence of internal necrosis made a malignant process extremely unlikely. Additionally, initial flow cytometry, biopsy, and EUS-guided FNA were also negative for malignancy. Therefore, at this point, alternative considerations other than malignancy were being considered, such as an organizing hematoma, given the findings described above.

On further questioning, the patient recalled cliff diving from 70 feet, five months earlier. This raised suspicion of trauma-induced retroperitoneal hematoma with secondary IVC compression and thrombosis.

The patient underwent catheter-directed thrombolysis and thrombectomy. He was transitioned to therapeutic anticoagulation with low molecular weight heparin. Repeat venogram showed reduced thrombus burden.

Outcome and follow-up

At one week, his leg swelling resolved. One-month imaging showed hematoma and thrombus regression. He resumed biking but experienced pain while running. Due to ongoing claudication, he underwent endovascular recanalization. At three months, he remained asymptomatic on anticoagulation.

## Discussion

Clinical history plays a vital role in identifying risk factors and underlying causes of IVCT. Relevant history includes previous DVT, malignancy, surgery, immobility, and thrombophilia [1,2,5]. In young patients, congenital anomalies should be suspected. Abdominal trauma, catheterization, or surgery may suggest acquired causes [1,2,5]. A focused and thorough history can guide appropriate imaging, expedite diagnosis, and inform individualized management strategies.

Blunt traumatic inferior vena cava injury (BTIVCI) is rare, reported in 1-3.2% of trauma cases [6]. The IVC’s retroperitoneal location offers some protection; however, deceleration trauma can cause vessel disruption, particularly in the retrohepatic or suprahepatic segments, which are associated with high mortality [6].

External compression of the IVC from hematoma or mass can promote venous stasis and thrombosis. In this case, the patient's cliff diving incident likely caused a retroperitoneal hematoma, compressing the IVC and predisposing to thrombosis and collateral formation, as evidenced by imaging. This case is unique in that it illustrates a rare instance of IVCT secondary to blunt abdominal trauma from cliff diving.

There are currently no definitive guidelines for IVCT management. Anticoagulation is standard for nontraumatic IVCT [4]. Catheter-directed thrombolysis (CDT) or pharmacomechanical catheter-directed thrombolysis (PMCT), combined with angioplasty or stenting, are additional options, especially in acute or subacute cases [4]. Chronic cases (>28 days) may benefit more from percutaneous transluminal angioplasty or stenting, with limited utility for thrombolysis [4].

## Conclusions

IVCT, though rare, carries a significant risk and requires high clinical suspicion. A thorough patient history is critical for identifying underlying risk factors and guiding diagnostic evaluation. Notably, high-impact sporting activities should be considered potential contributors to trauma-related IVCT. Imaging modalities such as CT or MRI are essential for accurate diagnosis and anatomical assessment. A full thrombophilia panel should be considered in younger patients without an obvious cause. Anticoagulation remains the cornerstone of therapy, with endovascular options for selected cases.
